# Brightness masking is modulated by disparity structure

**DOI:** 10.1016/j.visres.2015.02.010

**Published:** 2015-05

**Authors:** Vassilis Pelekanos, Hiroshi Ban, Andrew E. Welchman

**Affiliations:** a School of Psychology, University of Birmingham, UK; b Department of Psychology, University of Cambridge, UK; c Center for Information and Neural Networks, National Institute of Information and Communications Technology, Osaka, Japan; d Graduate School of Frontier Biosciences, Osaka University, Osaka, Japan

**Keywords:** Brightness estimation, Visual masking, Binocular depth perception, Filling-in

## Abstract

The luminance contrast at the borders of a surface strongly influences surface’s apparent brightness, as demonstrated by a number of classic visual illusions. Such phenomena are compatible with a propagation mechanism believed to spread contrast information from borders to the interior. This process is disrupted by masking, where the perceived brightness of a target is reduced by the brief presentation of a mask (Paradiso & Nakayama, 1991), but the exact visual stage that this happens remains unclear. In the present study, we examined whether brightness masking occurs at a monocular-, or a binocular-level of the visual hierarchy. We used backward masking, whereby a briefly presented target stimulus is disrupted by a mask coming soon afterwards, to show that brightness masking is affected by binocular stages of the visual processing. We manipulated the 3-D configurations (slant direction) of the target and mask and measured the differential disruption that masking causes on brightness estimation. We found that the masking effect was weaker when stimuli had a different slant. We suggest that brightness masking is partly mediated by mid-level neuronal mechanisms, at a stage where binocular disparity edge structure has been extracted.

## Highlights

• Brightness masking is affected by three-dimensional configuration.
• Masks with the same 3-D orientation yield a greater masking effect.
• Brightness estimation is likely to be partially mediated by mid-level mechanisms.

## 1 Introduction

The perceived brightness of a surface differs substantially from its photometric luminance. A number of classic visual illusions demonstrate the important role that contrast edges play in the visual appearance of an enclosed surface. For instance, when viewing the Craik–O’Brien–Cornsweet illusion, observers interpret isoluminant areas as having different brightness due to the luminance intensity ramps at their edges. The spatial influence of such contrast edge effects can be extensive (for example, Adelson, 2000, Komatsu, 2008).

Such phenomena can be understood in terms of the operation of spatial filtering processes that act at very early stages (pre-cortical) of visual processing (Blakeslee and McCourt, 1999, McArthur and Moulden, 1999, Otazu et al., 2008, Watt and Morgan, 1985). Alternatively, higher-level explanations have been offered on the basis that the brain employs propagation mechanisms (“filling-in”), whereby attributes encoded at one portion of the scene (e.g., contrast edges) influence the perceptual appearance of stimulus attributes that the visual system appears less ready to encode (e.g., regions of homogenous intensity) or unable to sense (e.g., due to the retinal blind spot) (Anstis, 2010, Komatsu, 2006, Pessoa et al., 1998). Electrophysiological recordings from the visual cortex provide some support for the notion that neural activity spreads across the cortex during presentation of displays that involve filling-in effects (De Weerd et al., 1995, Lamme et al., 1999). This lateral spreading of activity may provide part of explanation for the absence of ‘missing’ information in our perceptual interpretation of the world, and is compatible with psychophysical evidence for the lateral spread of contrast information across the cortical surface (Davey, Maddess, & Srinivasan, 1998).

One means of studying the mechanisms of brightness perception is to interfere with the putative filling-in mechanisms that may support it. Paradiso and Nakayama (1991) developed such an approach using metacontrast masking, reasoning that if brightness estimation involves the spread of activity from the border of a surface towards its interior, then it should be possible to interrupt it. Specifically, they hypothesized that if contrast information is propagated from contrast edges, the subsequent presentation of new border signals should interfere with the filling-in process before it was completed. They found that the brightness at the centre of a uniform target was considerably reduced when followed (50–100 ms) by a briefly presented circular mask (concentric with the target). Moreover, they observed a trade-off between the distance between the edges of the target and mask and the time at which the mask had a suppressive effect on brightness, which they suggested was compatible with a filling-in process where spreading of activity occurred at around 130 deg/s. They further observed that masking was greater under dichoptic presentation (target and mask presented to different eyes) than under monoptic presentation: in the former case, dramatic brightness suppression occurred even with simultaneous presentation of target and mask. This indicates that binocular processes are involved in the estimation of brightness, indicating contributions at the cortical level, although effects of rivalry or binocular summation could not be separated.

Information about three-dimensional scene structure has previously been suggested to be important for brightness estimation. For instance, computational models of early visual processing and brightness estimation (Grossberg and Mingolla, 1985, Grossberg and Todorovic, 1988) posit a role for disparity signals in constraining filling-in mechanisms for brightness (Kelly & Grossberg, 2000). Moreover, high-level theories of brightness (Adelson, 1993) and lightness (e.g., Anderson and Winawer, 2005, Gilchrist, 1977, Knill and Kersten, 1991) incorporate information about the three-dimensional scene structure that is available from the image.

Here we sought to test the contribution of disparity-defined three-dimensional scene information in guiding the impression of brightness by employing a modified version of the paradigm developed by Paradiso and Nakayama. In particular, we asked whether the brightness reduction induced by a mask was affected by the depth configuration of the target and mask. We reasoned that if brightness estimation takes place at a low level of processing (i.e. before depth estimation has occurred) we would find no change in the effect of a briefly presented mask when the mask and target had the same or opposite disparity-defined slants. However, if brightness estimation involves binocular disparity edge information, we anticipated that masking would be greatest when the target and mask where spatially coincident. In our first experiment we considered the effects of opposite slants for the target and mask. In experiment two, we then examined the sensitivity of the masking effect to gradations of slant differences between the target and mask.

## 2 Methods

### 2.1 Participants and apparatus

Eleven participants (including authors H.B. and V.P.) took part in Experiment 1 (mean age = 27.7, *SD *= 4.58; 3 females) and nine in Experiment 2 (mean age = 27.4, *SD *= 4.67; 1 female). All participants except the authors were naïve to the purpose of the study and were recruited from staff and students at the University of Birmingham and the University of Cambridge. All had normal or corrected to normal vision, and provided written informed consent. They were screened to ensure they could reliably discriminate depth positions defined by at least 1 arcmin of horizontal disparity. The protocols for the experiment were approved by the University of Birmingham’s STEM ethics committee. The work was carried out in accordance with the Code of Ethics of the World Medical Association (Declaration of Helsinki).

Stimuli were viewed through a mirror stereoscope, where the two eyes viewed separate gamma corrected CRT (ViewSonic FB2100X) monitors from a distance of 50 cm. Screen resolution was 1600 × 1200 pixels at 100 Hz. Luminance calibration was achieved by linearizing grey-level values using a Minolta LS110 photometer. Presentation monitors were recalibrated regularly to ensure that stimulus luminance was constant for different participants and across experiments.

### 2.2 Stimuli and procedure

**Fig. 1:**
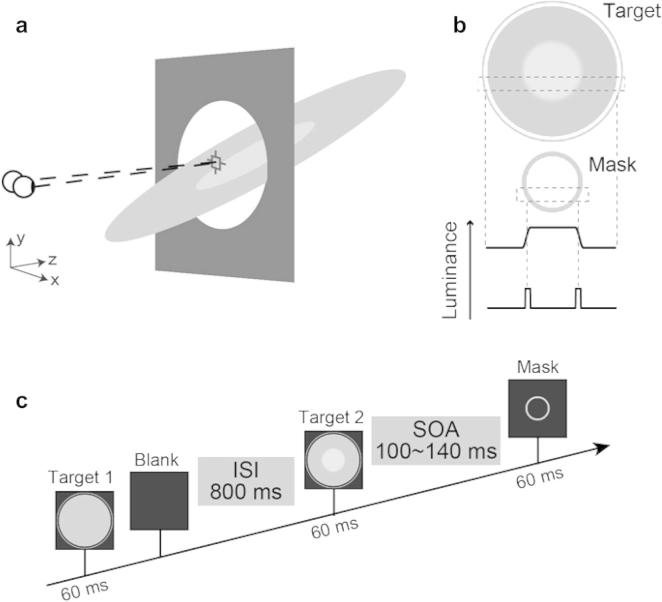
(a) A cartoon illustration of the target and its depth configuration. The target and masking stimuli were slanted in depth around a horizontal axis. (b) Illustration of the luminance profiles for the ‘test’ interval stimuli presented in the study. (c) The time sequence of stimulus presentation on a typical trial. Stimuli are depicted in the frontal plane for ease of representation; however for Experiment 1 both target and mask stimuli were slanted, and for Experiment 2 the masks were slanted in depth.

The stimuli were circular target disks (diameter = 12°) and a mask which was an unfilled circle (diameter = 5.2°; line width = 0.4°) (Fig. 1). One of the targets (the reference; ‘target 1’ in Fig. 1c) was a uniform disk (luminance of 101.7 cd/m^2^) while the other target (the test; ‘target 2’ in Fig. 1c) had a centre-surround configuration with a blurred interior boundary (see Fig. 1b). The diameter of the centre portion was 5.2°, and we applied blur to the boundary using a 2-D Gaussian-kernel of FWHM = 0.2°. The luminance of the centre portion of the disk in the test target was controlled by an adaptive staircase and varied from 101.7 to 135 cd/m^2^; the surround had a constant luminance of 101.7 cd/m^2^.

Prior to taking part in the experiment, participants were dark adapted for 5 min, followed by two minutes of passive viewing on a mid-level grey patch of 67.8 cd/m^2^ (this corresponded to the background luminance during stimulus presentation). Brightness judgments were measured using a two interval forced choice paradigm where the inter-stimulus interval (ISI) was 800 ms. During the reference interval, a single disc with a uniform luminance of 101.7 cd/m^2^ was presented for 60 ms. During the test interval, a target disc (with variable luminance at its centre) was followed by the mask after a pre-defined time interval (stimulus onset asynchrony – SOA). The order of the reference and test stimulus presentation was randomised. We measured luminance increment thresholds, defined as the just noticeable difference. In particular, participants judged whether the first or the second target had a brighter centre. Thresholds were calculated using the QUEST staircase method (Watson & Pelli, 1983) to obtain the 82% threshold. Luminance was decreased after three successive correct responses, but increased after one incorrect response (i.e., 3-up and 1-down staircase).

### 2.3 Masking properties

For the test interval presentations, a mask was presented after the target stimulus (metacontrast backward masking; see Breitmeyer and Ogmen, 2000, Breitmeyer and Ogmen, 2006). The mask was centred on the target, and had the same diameter (5.2°) as the centre portion of the target. The target and the mask remained on screen for 60 ms each, while the exact interval (SOA) between them was tailored to individual participants (see Section 2.4 below).

### 2.4 Stimulus onset asynchrony (SOA) estimation

Prior to taking part in the main experiments, participants completed a session designed to estimate their SOA threshold. It is known that masking is a function of the SOA (Alpern, 1953), with little masking at either very short or long SOAs, but dramatic reductions in target’s visibility in-between. Paradiso and Nakayama (1991) tested the influence of SOA on brightness masking finding maximal effects for an SOA of 50–100 ms. Other studies on backward masking find SOA time-windows for optimal target suppression vary in the range 30 and 150 ms (Breitmeyer and Ogmen, 2006, Green et al., 2005, Polat et al., 2007), with differences between individual participants. We therefore chose to tailor the maximal masking effect by identifying optimal values for each participant.

This testing session consisted of three blocks of 50 trials. Stimuli were orientated in the fronto-parallel plane. The participants’ task was to indicate which interval had the brighter centre, and we estimated increment thresholds that gave the maximum masking effect using the QUEST method. We found that estimated SOA thresholds for two participants exceeded 250 ms, which is outside the range expected for genuine metacontrast masking. We retested these (naïve) participants in a second session and again found SOA thresholds in excess of 250 ms. We therefore excluded them from further study. For the remaining nine participants, the mean estimated SOA was 116.7 ms (*SEM* = 4.4).

### 2.5 Main experimental conditions

**Fig. 2:**
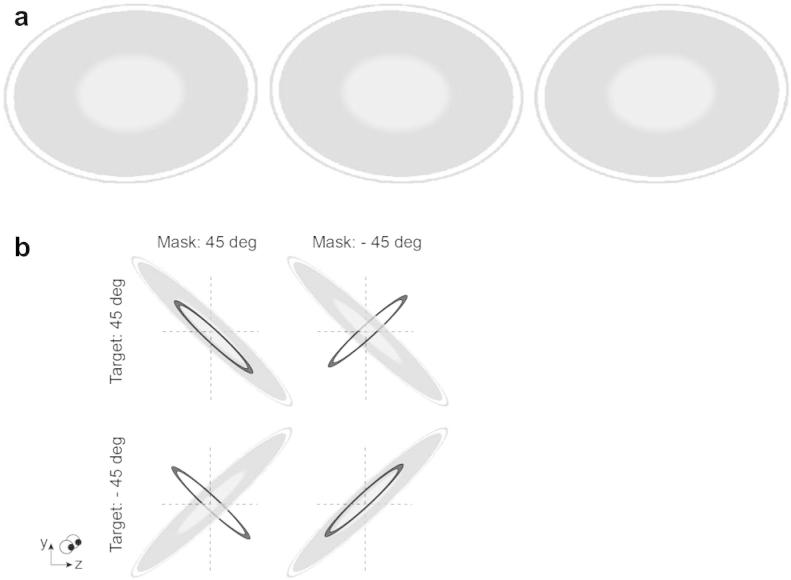
(a) Stereogram illustrating a sample of the monocular views of the stimuli of experiment 1. (b) Illustration of the target and mask configurations for Experiment 1. The control experiment consisted of the same conditions, but the stimuli had no binocular disparity/3-D information, since each eye viewed the same stereo half image.

In Experiment 1 we tested how the relationship between the slant of the target and the mask affected brightness estimation. We presented targets and masks that were slanted in depth (±45°) with respect to the fronto-parallel plane (Fig. 2a provides stereograms for cross-fusion). We varied whether the target and the mask had the same or opposite slants, resulting in four different stimulus configurations (Fig. 2b). We measured thresholds by averaging over ten blocks (three participants) or four blocks (six participants) of 200 trials each. The different conditions were randomly interleaved during an individual run.

To ensure that any possible differences in masking were due to the slant of the surface, we included a control condition in which we measured masking for binocular presentation of the images viewed by the left eye in the main experiment 1. In particular, the disparity applied to the stimuli would create small offsets between the edges of the mask and target when they had binocularly specified opposite slants while edges would be aligned for stimuli with the same slant. Thus, we measured whether differential masking caused by these small, monocularly available signals might affect masking for ‘same’ and ‘opposite’ slant conditions. As the identical images were presented to both eyes in this condition, the stimuli had no binocular disparity and appeared flat. Thresholds were calculated as mean performance over ten blocks for three observers and over four blocks for six observers.

## 3 Results

### 3.1 Experiment 1

**Fig. 3:**
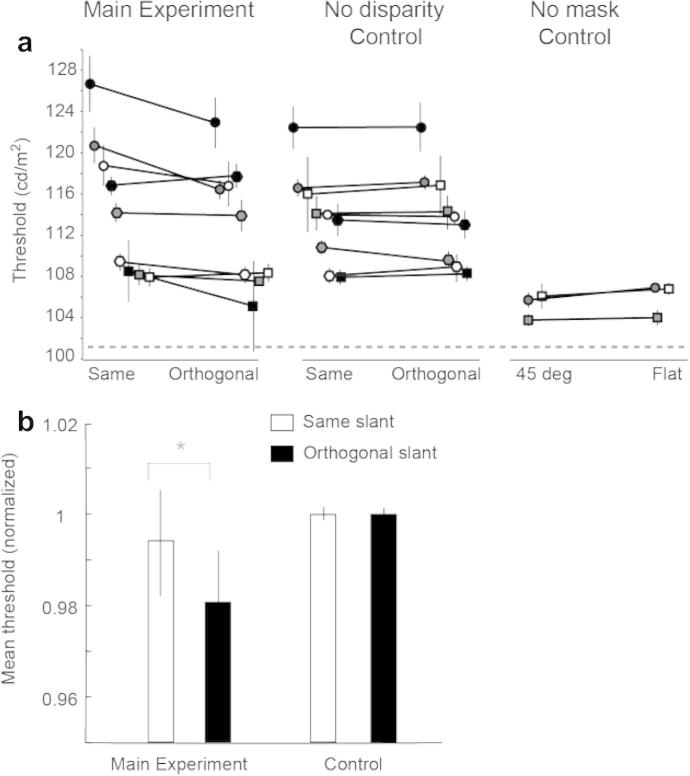
(a) Raw discrimination thresholds across the ‘same’ and ‘orthogonal’ stimuli configurations for the main and the no-disparity control experiments for all nine participants (left panels). Raw thresholds for the three additional participants across the 45° slants and the flat conditions for the no-masking control (right panel). The dotted continuous line represents the constant luminance of the reference target surface whose brightness was compared with the test target throughout the experimental conditions. Individual participants are indicated by each plotting symbol; black and grey filled circles represent data from authors HB and VP. (b) Average discrimination thresholds as a function of stimulus configurations for the main experiment and the no-disparity control conditions. Threshold values here are normalised by dividing each luminance (cd/m^2^) value by the mean luminance measured in the control conditions, for each participant. Statistically significant differences are indicated by an asterisk. Error bars show the between-subjects standard error of the mean.

In experiment 1, we were interested in examining whether brightness masking would be stronger when the target and mask shared the same 3-D orientation. We measured just noticeable difference thresholds for the central portion of the target disc. Raw (luminance measured in cd/m^2^) data for each participant are shown in Fig. 3a. Although there is variability between individuals, these data reveal that for 7 out of the 9 participants, thresholds were elevated (stronger brightness masking) in the ‘same’ conditions, where the target and the mask shared 3D orientations, compared to the orthogonal conditions. In order to remove the variability in overall thresholds between participants, we normalised (per participant) the data by dividing each luminance threshold for the experimental conditions by the mean luminance thresholds measured in the no-disparity control conditions.

The average (normalised) discrimination thresholds from Experiment 1 are shown in Fig. 3b. We found that thresholds were higher in the conditions where the target and mask had the same slant orientations, compared to the conditions where their slants were orthogonal. In particular, using a 2 (slant congruence: same vs. orthogonal) × 2 (slant sign: positive vs. negative) repeated-measures ANOVA we found that there was a main effect of target-mask congruence (*F*(1, 8) = 6.06, *p* = .039), but no effect of slant sign (*F*(1, 8) = 1.35, *p* = .278) and no interaction (*F*(1, 8) < 1, *p* = .730).

Considering data from the control condition in which there was no difference in the slant of the target and mask (the stimuli appeared frontoparallel), we found no effect of slant congruence on the extent of masking (*F*(1, 8) < 1, *p* = .935), nor slant sign (*F*(1, 8) < 1, *p* = .881) or an interaction (*F*(1, 8) < 1, *p* = .935). This suggests that the masking effect observed in the stereoscopically defined condition could not be attributed to subtle differences in the monocular images, but rather is due to the extraction of binocular signals.

In addition, to evaluate the effectiveness of masking *per se* in our paradigm, we conducted a control, no-masking, experiment on three participants. We preserved the standard experimental configurations of the target (−45 and 45° slants) and also presented a flat condition (target appearing with zero slant), where none of the targets/conditions was followed by mask. The mean increment thresholds in this no-masking control was 105.4 cd/m^2^, i.e., the just noticeable difference (jnd) was 3.7 cd/m^2^ (reference target’s luminance being 101.7 cd/m^2^). In contrast, in the main experiment, the mean threshold was 114.2 cd/m^2^ (jnd of 12.5 cd/m^2^).

Inspecting Fig. 3a and comparing the results from the ‘same’ condition in the main experiment (±45° slant) and the ‘same’ condition in the no-disparity control condition (frontoparallel stimuli), might suggest that brightness masking is slightly higher for slanted targets. However, there was no reliable difference between the two conditions (mean threshold in main condition = 114.2 cd/m^2^ vs. 114.9 cd/m^2^ in the control). A repeated-measures ANOVA confirmed that there were no statistically significant differences (*F*(1, 8) < 1, *p* = .57).

### 3.2 Experiment 2

**Fig. 4:**
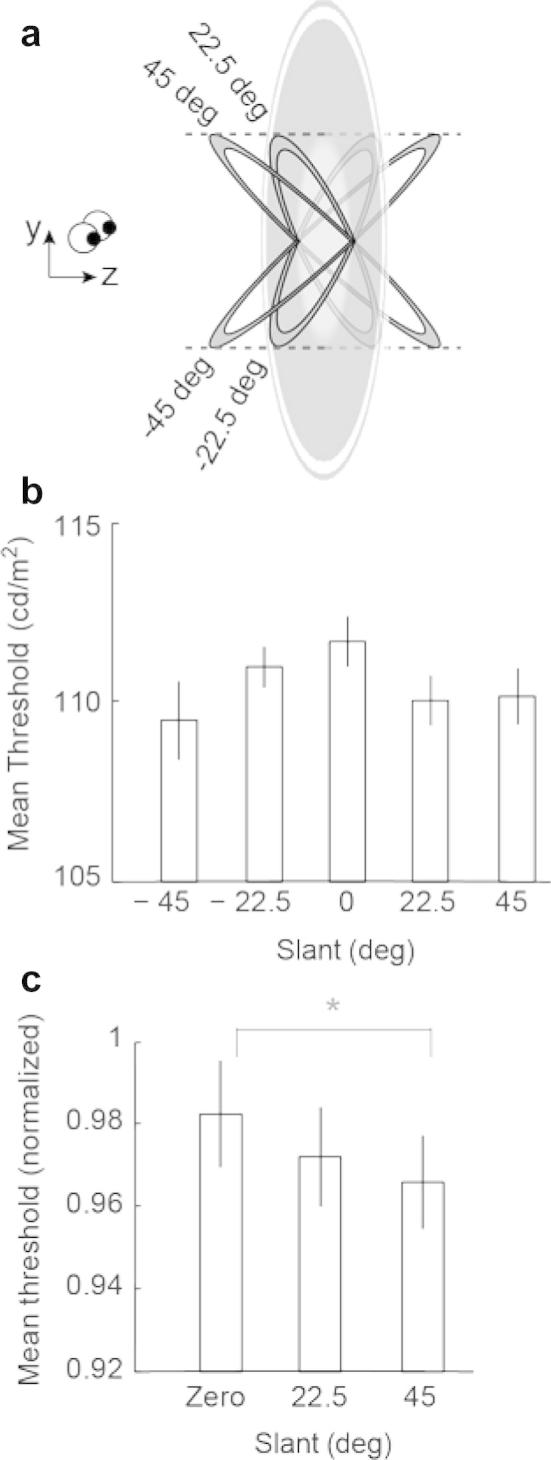
(a) Illustration of the stimulus configuration for experiment 2. The target had zero disparity, appearing flat, across all the conditions. The mask had different slants with respect to the target (0, ±22.5 and ±45°) resulting in five target-mask configurations. As the figure shows, we controlled the spatial extent of the mask so that it covered the same spatial extent irrespectively of the slant. (b) Between-subjects average thresholds in the five individual experimental conditions expressed in measured luminance. (c) Average normalised thresholds data for the different slant levels. Statistically significant differences are indicated by an asterisk. Error bars show the between-subjects standard error of the mean.

In our second experiment we sought to determine whether the mask interferes with the target only when stimuli share the same slant, or whether interference occurs when the target and mask are slightly misaligned. We therefore presented a frontoparallel (0° angle) target stimulus, and then masks of different slant angles with respect to it (0, ±22.5 or ±45°; Fig. 4a). We measured thresholds by averaging over four blocks of 250 trials each, while conditions in each block were randomly interleaved. A repeated-measures ANOVA showed that masking was stronger (higher thresholds; see Fig. 4b) when the mask and target shared the same frontoparallel orientation compared to the slanted conditions of the mask [*F*(4, 32) = 5.12, *p* < .01].

We normalised the data to remove between participant variability (as described for Experiment 1), and grouped opposing slant values (Fig. 4c). We found an effect of slant on brightness masking: a one-way ANOVA (with 45, 22.5 and 0° conditions as factors) showed a significant effect of magnitude [*F*(2, 16) = 8.87, *p* < .01]. Post-hoc analysis using Bonferroni correction for multiple comparisons indicated a significant difference between zero and 45° slant (*p* < .01), but not between zero and 22.5° (*p* = .087), or between 45 and 22.5° conditions (*p* = .557). These results indicate that masking is weakest (decreased thresholds) when the target and mask are spatially misaligned (45°).

## 4 Discussion

In the present study, we investigated whether brightness estimation incorporates the 3-D information of disparity-defined slanted surfaces. We used a masking paradigm to examine whether the disruption of brightness estimation from backward masking is modulated by the slant configurations of target and mask stimuli. We report an influence of surface slant on brightness masking. Specifically, in experiment 1, we found targets and masks that shared the same 3-D orientation produced a greater attenuation of brightness. Moreover, a no-disparity control condition indicated that this difference could not be explained by subtle image differences at the monocular level. Experiment 2 examined how sensitive this effect was to the precise 3-D orientation of the masking surfaces. While we found an influence of mask orientation on brightness, we did not observe a tight tuning of the effect to the precise slant angle. More generally, it is important to note that the modulation of masking by changes in surface slant was small. Thus, it is likely that brightness estimation involves a substantial monocular component, with a relatively minor contribution from disparity-defined surface structure information. These results suggest that brightness estimation is at least partially mediated by mid-level neuronal mechanisms where disparity edge signals have been extracted.

While our approach is grounded in the work of Paradiso and Nakayama (1991), it is important to note differences existed in the stimulus configuration between the two studies: Paradiso and Nakayama used uniform surfaces and asked participants to make brightness matches using the method of adjustment. Here, we used a centre-surround configured test target (‘target 2’ in Fig. 1c) whose central area, without masking, appeared brighter than the surround. We asked participants whether this central area was brighter than the corresponding area of the reference target (‘target 1’ in Fig. 1c). The test target was followed by a mask, whereas the reference was followed by no mask and also had uniform and constant luminance. Thereby we sought to use a staircase procedure to quantify the masking effects. Our approach of using a bipartite stimulus may be responsible for the relatively modest masking effects we observe in this study. Paradiso and Nakayama reported masking effects that could approach two orders of magnitude (although their stimuli were considerably more luminous than we could achieve on our setup). It is possible that backwards masking was weaker in our study because brightness propagation could have started inside the masked region from the central portion of the disk. Our use of blurred boundaries between the two portions of the disk was intended to attenuate any such effect, and it is important to consider that while this effect may have been present at the start of an experimental session (i.e., large luminance difference between centre and surround), the contribution of an interior boundary signal would be considerably reduced as the luminance contrast between the centre and surround was adaptively reduced by the staircase algorithm.

Our data suggest that binocular disparity edges modulate the degree of disruption that backward masking causes to the estimation of a surface’s brightness. Apparent brightness has been strongly associated with a propagation (‘filling-in’) process. Previous work suggests that active filling-in processes are unlikely to explain the perceptual filling-in of motion and depth information (Welchman & Harris, 2003) given lower spatial resolution of these signals. Nevertheless, the current study suggests a modest role for disparity-defined edge structure in modulating brightness estimation. This can be framed within the framework of Grossberg (1994) model according to which, once the boundaries of surfaces are registered, the 3-D surfaces are filled-in/generated at a stage not earlier than area V4.
